# Predictive factors for successful pregnancy in an egg-sharing donation program

**DOI:** 10.5935/1518-0557.20190087

**Published:** 2020

**Authors:** Daniela Paes de Almeida Ferreira Braga, Amanda Souza Setti, Assumpto Iaconelli Jr., Edson Borges Jr.

**Affiliations:** 1 Fertility Medical Group, São Paulo, SP, Brazil; 2 Instituto Sapientiae – Centro de Estudos e Pesquisa em Reprodução Assistida, São Paulo, SP, Brazil

**Keywords:** egg donation, oocyte donor, vitrification, implantation, pregnancy

## Abstract

**Objective:**

To investigate the predictive factors for successful pregnancy in oocyte recipient ICSI cycles in an egg-sharing donation program.

**Methods:**

Analysed data were obtained via chart review of 1505 vitrified oocytes donated from 268 patients to 225 oocyte recipients, undergoing 307 ICSI cycles. Patients were participating in an egg-sharing donation program between January 2015 and May 2017. Adjusted generalised linear models were used to investigate the impact of oocyte donor and recipient characteristics on recipients’ pregnancy achievement.

**Results:**

Implantation rate in the oocyte donor was highly correlated with pregnancy achievement in the oocyte recipient’s cycles (ExpB: 1.181, CI: 1.138-1.226, *p*<0.001). The ROC curve analysis demonstrated that the implantation rate in the oocyte donor has a strong predictive value for pregnancy success in the oocyte recipient (area under the curve: 0.98, CI: 0.95-0.99, *p*<0.001). Pregnancy in oocyte donors and recipients were highly associated (ExpB: 54.6, CI: 28.1-105.8, *p*<0.001), regardless of the oocyte recipient’s age. In oocyte recipients, the high-quality embryos rates on days 2 (ExpB: 3.397, CI: 1.635-7.054, *p*=0.001) and 3 (ExpB: 6.629, CI: 1.185-37.092, *p*=0.031), and blastocyst development rates (ExpB: 2.331, CI: 1.086-5.001, *p*=0.030) were positively associated with pregnancy outcome.

**Conclusion:**

The strong association in pregnancy success between donors and recipients, and the lack of correlation between donor characteristics and cycles’ outcomes, demonstrate the power of oocyte quality on the success of ICSI treatment.

## INTRODUCTION

Since the first birth from an oocyte fertilised in vitro and transferred to a recipient was reported (Lutjen *et al*., 1984), advances in assisted reproductive technologies (ART) have allowed women who cannot use their own oocytes to achieve a viable pregnancy through oocyte donation. The success of oocyte donation is reportedly influenced by multiple factors including the age of the oocyte donor and recipient, the embryo quality, endometrial receptivity, and others (Yeh *et al*., 2014; Provost *et al*., 2016; Labarta *et al*., 2017; Capelouto *et al*., 2018).

Donor age is one of the most significant factors affecting in vitro fertilisation (IVF) outcomes (Stolwijk *et al*., 1997; Tufan *et al*., 2004). In fact, advancing age not only leads to declining fertility by reduced oocyte quantity, but also due to reduced oocyte quality (Baird *et al*., 2005). Although the effect of increased maternal age on reproduction has been widely studied, the influence of the oocyte donor’s age on the recipient’s pregnancy success is still controversial. While many investigators have reported that recipient age is inversely related to oocyte donation success (Levran *et al*., 1991; Check *et al*., 1993; Meldrum, 1993; Borini *et al*., 1996; Marcus *et al*., 2016), others have not observed this relationship (Abdalla *et al*., 1997; Paulson *et al*., 1997; Remohi *et al*., 1997).

The low variability of age and embryo quality in oocyte donation programs gives the opportunity for examination of the independent effect of uterine receptivity on implantation outcomes. The endometrium is a highly dynamic tissue that undergoes cyclic cellular proliferation, differentiation, and immune cell trafficking in response to changing circulating ovarian-derived steroids (Valdes *et al*., 2017).

Accumulated evidence has suggested that there is an alteration of endometrial receptivity in patients with recurrent implantation failure (RIF) (Li *et al*., 1993; Demirol & Gurgan, 2004; Margalioth *et al*., 2006). Despite this, the role of the endometrial thickness and pattern as predictors of success in oocyte donation cycles is still controversial (Abdalla *et al*., 1994; Zenke & Chetkowski, 2004; Barker *et al*., 2009; Oyesanya *et al*., 2009; Amui *et al*., 2011; Dain *et al*., 2013; Mac Conell *et al*., 2015; Zhang *et al*., 2018).

Previous studies have suggested that the body mass index (BMI) likely plays a role in the poorer endometrial receptivity in oocyte donation programs. Increasing oocyte recipient BMI was associated with a reduction in clinical pregnancy and live birth rates (Bellver *et al*., 2007; 2013; Provost *et al*., 2016). On the other hand, (Luke *et al*., 2011) found that increasing BMI was associated with a significant rise in failure to achieve pregnancy with the use of autologous oocytes, but no difference with the use of donor oocytes was observed. Moreover, a systematic review and meta-analysis suggested no effect of recipient BMI on the chance of pregnancy (Jungheim *et al*., 2013).

The same lack of consensus is observed concerning the association of other donor characteristics such as response to controlled ovarian stimulation (Barton *et al*., 2010; Baker *et al*., 2015; Hariton *et al*., 2017) in recipients’ pregnancy achievement. Therefore, the goal for the present study was to investigate the predictive factors for successful pregnancy in oocyte recipient intracytoplasmic sperm injection (ICSI) cycles in an egg-sharing donation program.

## MATERIALS AND METHODS

### Patients and experimental design

Analysed data were obtained via chart review of 307 ICSI cycles with 1505 vitrified oocytes donated from 177 patients to 225 oocyte recipients in a private university-affiliated IVF center. Patients were participating in an egg-sharing donation program between January 2015 and May 2017. For this sample size, computed achieved post-hoc power was 100%, considering pregnancy achievement as the main outcome measure.

Oocyte donors were between the ages of 19 and 34 years, and recipients were between the ages of 26 and 50 years. The impact of oocyte donor characteristics (age, BMI, number of follicles, retrieved oocytes, total dose of FSH administered for COS, estradiol peak, pregnancy rate and implantation rate) and recipient characteristics (age, BMI, endometrial thickness, fertilisation rate, high-quality-embryos rate on cleavage stage, and blastocyst formation rate) on recipients’ pregnancy achievement was evaluated.

All patients signed a written informed consent form, and the study was approved by the local institutional review board.

### Controlled ovarian stimulation and laboratory procedures

Controlled ovarian stimulation was performed using recombinant follicle-stimulating hormone (r-FSH, Gonal-F^®^; Serono, Geneva, Switzerland), with pituitary blockage using a gonadotropin-releasing hormone (GnRH) antagonist, cetrorelix acetate (Cetrotide^®^; Merck KGaA, Serono, Geneva, Switzerland).

Follicular growth was monitored using transvaginal ultrasound examination starting on day 4 of gonadotropin administration. When adequate follicular growth and serum estradiol levels were observed, leuprolide acetate (Lupron^®^; TAP Pharmaceuticals, North Chicago, IL, United States) was administered to trigger the final follicular maturation. The oocytes were collected 35 hours later through transvaginal ultrasound ovum pickup.

The recovered oocytes were assessed to determine their nuclear status. Those in metaphase II were vitrified if destined for the recipients, or submitted to ICSI if destined for the oocyte donors.

### Oocyte vitrification and warming

The vitrification and warming procedures were performed using the Cryotop method (Kuwayama *et al*., 2005). Within three hours after ovum pickup, vitrification was achieved by the initial exposure of the oocytes to the equilibration solution, followed by a 30-second exposure to the vitrification solution.

Individual oocytes were then picked up in an extremely small volume (<0.1 mL) of vitrification solution, to facilitate rapid cooling, and placed on top of a very fine polypropylene strip attached to a hard-plastic handle. As soon as the oocyte was placed onto the thin polypropylene strip of the Cryotop, it was immediately submerged vertically into liquid nitrogen. Then the thin strip was covered with a hard-plastic cover on top of the Cryotop sheet.

For warming, the protective cover was removed from the Cryotop while it was still submerged in liquid nitrogen, and the polypropylene strip of the Cryotop was immersed directly into the thawing solution at 37°C for 1 minute. Oocytes were retrieved and transferred into dilution solution for 3 minutes and then washed twice in the washing solution for 5 minutes each.

The tools and solutions required for the vitrification and warming processes were obtained from Kitazato^®^ (Tokyo, Japan).

Within three hours after warming, oocytes were submitted to ICSI following routine procedures (Palermo *et al*., 1997).

### Embryo culture and morphology evaluation

Approximately 16 hours after ICSI, fertilisation was confirmed by the presence of two pronuclei and the extrusion of the second polar body. The embryos were maintained in a 50 µL drop of culture medium (Global^®^; LifeGlobal, CT, USA) covered with paraffin oil in a humidified 6% CO_2_ atmosphere at 37°C for three days.

The embryo morphology was assessed 16-18 h post-ICSI and on the mornings of days two, three, and five of embryo development using an inverted Nikon Diaphot microscope (Eclipse TE 300; Nikon, Tokyo, Japan) with a Hoffmann modulation contrast system under 400X magnification.

To evaluate the cleavage-stage morphology, the following parameters were recorded: the number of blastomeres, the percentage of fragmentation, the variation in blastomere symmetry, the presence of multinucleation, and the defects in the zona pellucida and cytoplasm. The high-quality cleavage-stage embryos were defined as those with all of the following characteristics: four cells on day two or 8−10 cells on day three, <15% fragmentation, symmetric blastomeres, the absence of multinucleation, colourless cytoplasm with moderate granulation and no inclusions, the absence of perivitelline space granularity and the absence of zona pellucida dysmorphism. Embryos lacking any of these characteristics were considered to be of low quality.

To evaluate the blastocyst morphology, embryos were given a numerical score from one to six based on their degree of expansion and hatching status as follows: 1, an early blastocyst with a blastocoel that was less than half of the volume of the embryo; 2, a blastocyst with a blastocoel that was greater than half of the volume of the embryo; 3, a full blastocyst with a blastocoel that completely filled the embryo; 4, an expanded blastocyst; 5, a hatching blastocyst; and 6, a hatched blastocyst. Full, expanded, hatching, and hatched blastocysts were classified as complete blastocysts.

Full blastocysts onwards, presenting morphologically normal inner cell mass (ICM) and trophectoderm (TE) were defined as high-quality blastocysts. A tightly packed ICM presenting many cells was defined as a high quality ICM. Similarly, the TE was classified as high quality by the presence of many cells forming a cohesive epithelium.

### Endometrial preparation

For donors, on the day after ovum pick-up, 600 mg of progesterone (Utrogestan^®^; Farmoquímica, Rio de Janeiro, Brazil) was vaginally administered per day. In the case of a positive β-hCG test, progesterone treatment was maintained until week 12 of gestation or was suspended in the case of a negative β-hCG test.

For recipients, after menses, endometrial development was followed by ultrasound examination, and the patients received 200 µg of transdermal 17-β oestradiol every 3 days (Estradot^®^; Noven Pharmaceuticals, Miami, USA).

Approximately 14 days after initiation of 17-β oestradiol administration, serum E2 levels and endometrial thickness were determined. When the endometrium showed proliferative morphology and thickness of at least 7.5 mm, 600 mg of progesterone (Utrogestan^®^; Farmoquímica, Rio de Janeiro, Brazil) was vaginally administered per day.

Both 17-β oestradiol and progesterone were administered concomitantly after embryo transfer and were suspended in the case of a negative β-hCG test. In the case of a positive β-hCG test, the 17-β oestradiol and progesterone treatments were maintained until weeks 6 and 12 of gestation, respectively.

### Embryo transfer and clinical follow-up

Embryo transfers were performed on day 5 of embryo development and one or two embryos were transferred per patient.

A pregnancy test was performed 10 days after embryo transfer. All women with a positive test had a transvaginal ultrasound scan 2 weeks after the positive test. A clinical pregnancy was diagnosed when the foetal heartbeat was detected. Pregnancy rates were calculated per transfer. The implantation rate was calculated by dividing the number of gestational sacs with foetal heartbeats by the number of transferred embryos. Miscarriage was defined as clinical pregnancy loss before 20 weeks.

### Data analysis and statistics

Computed achieved post-hoc power was 100%, considering pregnancy achievement as the main outcome measure. The calculation was performed using G*Power 3.1.7. Data are expressed as the mean ± standard deviation for continuous variables, while percentages are used for categorical variables. General Mixed Models (GMM) fit by restricted maximum likelihood was used to investigate the impact of oocyte donors’ and recipients’ characteristics on recipients’ pregnancy achievement.

Linear mixed effects models were generated using covariates as fixed effects and egg-donors and egg-recipients as random effects, with unstructured covariance structure. A Gaussian distribution was assumed, and we checked the normal distribution of model residuals to confirm goodness of fit. Final model selection was decided using Akaike’s Information Criterion (AIC) and Schwarz's Bayesian Criterion.

In a further step, a receiver operating characteristic (ROC) curve was constructed to investigate the predictive value of oocyte donor implantation rate on oocyte recipient pregnancy achievement.

The results are expressed as exponentiation of regression coefficient (ExpB), standard errors, 95% confidence interval (CI) and *p*-values. The ROC curve results are expressed as area under the curve (Gawecka *et al*., 2013) with 95% CI. A *p*<0.05 was considered statistically significant. Data analyses were conducted using the SPSS Statistics 21 (IBM, New York, NY, USA) and MedCalc Statistical Software version 16.4.3 (MedCalc Software bvba, Ostend, Belgium; https://www.medcalc.org; 2016).

## RESULTS

The donors’ and recipients’ characteristics are described in Table 1.

**Table 1:** Demographic and cycle characteristics for oocyte donors and recipients

Oocyte donors	
Age	30.1 ± 3.2 years
Body mass index	28.9 ± 3.7 kg/m^2^
Number of follicles	23.4 ± 13.7
Retrieved oocytes	18.7 ± 15.4
Total dose of FSH	2515.8 ± 675.1 IU
Estradiol peak	4376.1 ± 2372.2 pg/mL
Pregnancy rate (%)	120/255 (47.1)
Implantation rate	29.4 ± 40.1%
**Oocyte recipient**	
Age	41.7 ± 7.5 years
Body mass index	32.8 ± 4.3 kg/m^2^
Endometrial thickness	11.5 ± 2.3 mm
Fertilisation rate	81.4 ± 16.8%
High-quality embryos rate on D2	41.7 ± 45.8%
High-quality embryos rate on D3	36.2 ± 42,2%
Blastocyst formation rate	41.1 ± 38,3%
Pregnancy rate (%)	116/294 (39.5)
Implantation rate	29.6 ± 40.4%

Implantation rate in oocyte donors was highly correlated with pregnancy achievement in oocyte recipient cycles (ExpB: 1.181, CI: 1.138-1.226, *p*<0.001). Pregnancies in oocyte donors and recipients were highly associated (ExpB: 54.6, CI: 28.1-105.8, *p*<0.001), irrespective of oocyte recipient age. Oocyte donor age, body mass index, number of follicles, retrieved oocytes, total dose of FSH administered and estradiol peak were not associated with oocyte recipient pregnancy achievement (Table 2).

**Table 2:** Association between donors´ characteristics on oocyte recipient pregnancy achievement

Oocyte donor characteristics	Oocyte recipient pregnancy				
	B	ExpB	SE	CI	*p*
** Age **	0.022	1.023	0.033	0.958–1.092	0.502
** Body mass index **	-0.016	0.984	0.0373	0.915–1.059	0.665
** Number of follicles **	-0.012	0.988	0.0097	0.969–1.007	0.222
** Retrieved oocytes **	-0.0001	1.000	0.0082	0.984–1.016	0.988
** Total dose of FSH **	0.0004	1.000	0.0002	1.000–1.001	0.096
** Estradiol peak **	-0.00003	1.000	0.00006	1.000–1.000	0.642
** Pregnancy rate **	3.999	54.553	0.3381	28.122–105.824	<0.001
** Implantation rate **	0.166	1.181	0.0190	1.138–1.226	<0.001

B: regression coefficient;

SE: standard error;

ExpB: exponentiation of B;

CI: 95% confidence interval.

In oocyte recipients, no effect of the BMI, endometrial thickness and fertilisation rate on pregnancy success could be noted, but the high-quality embryos rates on days 2 (ExpB: 3.397, CI: 1.635-7.054, *p*=0.001) and 3 (ExpB: 6.629, CI: 1.185-37.092, *p*=0.031) and blastocyst development rates (ExpB: 2.331, CI: 1.086-5.001, *p*=0.030) were positively associated with pregnancy results. The age negatively influenced the pregnancy outcomes (Table 3).

**Table 3:** Association between recipients´ characteristics on oocyte recipient pregnancy achievement

Recipient characteristics	Oocyte recipient pregnancy				
	B	ExpB	SE	CI	*p*
** Age **	-0.056	0.945	0.0228	0.904–0.988	0.013
** Body mass index **	0.040	1.041	0.0264	0.988–1.096	0.132
** Endometrial thickness **	-0.054	0.947	0.0683	0.829–1.083	0.847
** Fertilisation rate **	0.007	1.007	0.0056	0.996–1.018	0.232
** High-quality embryos rate on D2 **	1.223	3.397	0.3729	1.635–7.054	0.001
** High- quality embryos rate on D3 **	1.140	6.629	0.3888	1.185–37.092	0.031
** Blastocyst formation rate **	0.846	2.331	0.3895	1.086–5.001	0.030

B: regression coefficient;

SE: standard error;

ExpB: exponentiation of B;

CI: 95% confidence interval.

**Figure 1:**
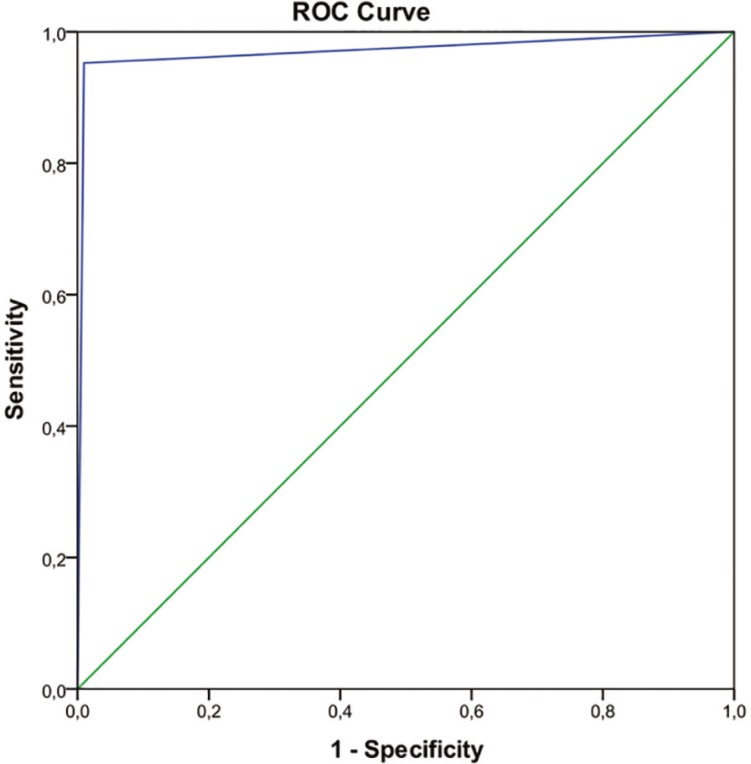
Receiver operating characteristic (ROC) curve for predicting recipients’ clinical pregnancy using donors’ implantation rate as test variable

The ROC curve analysis (Fig. 1) demonstrated that the implantation rate in oocyte donors has a strong predictive value for the achievement of pregnancy in oocyte recipients (area under the curve: 0.98, CI: 0.95-0.99, *p*<0.001).

## DISCUSSION

The use of IVF with donor oocytes has become an increasingly common treatment for women who are unable to conceive using their own oocytes. Data from the Society for Assisted Reproductive Technology (Luke *et al*., 2011) in the United States demonstrated that >9000 donor oocyte-recipient cycles were initiated in 2014 and 2015 (Dyer *et al*., 2016). However, considering the inconsistency of previously published reports, the analysis of factors which may predict the pregnancy outcome is needed. In the present study, the predictive factors of successful pregnancy in oocyte recipient cycles were investigated in an egg-sharing donation program. Oocyte donor implantation rate and successful pregnancy, high-quality embryos rate, and blastocyst development rate predict pregnancy achievement in the oocyte recipient cycle.

Intriguingly, the recipient’s, and not the donor’s, age negatively correlated with the pregnancy success. Donor age was supposed to be the most significant factor affecting IVF outcome. It is well established that female fertility decreases with increasing age as a reflection of not only declining oocyte quantity but also declining oocyte quality. In the present study, the reason why no correlation between the oocyte age and implantation could be noted may be due to the selection of oocyte donors, usually young women with a good prognosis, leading to low variability of age and oocyte quality.

As for the effect of the recipient’s age on a cycle’s outcome, it could be argued that the recipient’s age may be one of the factors implicated in the cross-talk between the embryo and the endometrium preceding implantation. Some studies found that recipient age has a negative impact on embryo implantation (Moomjy *et al*., 1999; Harris *et al*., 2002; Huang *et al*., 2008), while others reported that the age of the uterus does not affect embryo implantation rates, at least while cyclic hormonal stimulation and menstruation are present (Noci *et al*., 1995; Abdalla *et al*., 1997; Garcia-Velasco *et al*., 2003; Zenke & Chetkowski, 2004). However, some of these studies suggested that uterine receptivity is reduced for women over 40 years of age (Noci *et al*., 1995; Abdalla *et al*., 1997). In addition, the advanced age of the recipients was also associated with a significantly increased miscarriage rate (Huang *et al*., 2008).

The underlying factors that decrease endometrial receptivity in older women are still unclear; however, it has been suggested that the endometrium from these patients may undergo a reduction in progesterone receptors (Meldrum, 1993; Weckstein *et al*., 1993; Tapia-Pizarro *et al*., 2014).

Curiously, the recipients’ endometrial thickness did not influence the pregnancy rate. Endometrial thickness and pattern have been evaluated as possible predictors of pregnancy in multiple studies, with conflicting results. Some investigators have reported significant correlations between pregnancy rate and endometrial thickness or pattern (or both) (Al-Ghamdi *et al*., 2008; Zhao *et al*., 2012). Others have not shown such a relationship (Dietterich *et al*., 2002; Rashidi *et al*., 2005).

A recent meta-analysis showed that clinical pregnancy rates were significantly lower in women with endometrial thicknesses ≤7 mm at the end of ovarian stimulation (Kasius *et al*., 2014). Although Arce *et al*. (2015) reported that endometrial thickness ≥5 mm is a reasonable parameter for determining success in oocyte donation cycles, the best reproductive outcomes tend to be achieved if the endometrium is >8-9 mm (Dain *et al*., 2013).

According to Zhao *et al*. (2014), an endometrium with adequate growth, a triple-line on β-hCG day, or both, seems to be favourable for pregnancy. The endometrial thickness, however, is not prognostically useful in predicting the occurrence of pregnancy. In addition, for the present study, recipients were administered hormone replacement therapy for endometrial preparation, and apparently the endometrial thickness in artificially prepared cycles does not seem to influence pregnancy outcome. Noteworthy is that only a very small proportion of cycles demonstrated a thin endometrium after artificial preparation.

Our results also showed that the embryo quality in the cleavage stage, the blastocyst formation, and donor clinical outcomes are positively correlated with pregnancy success. was here We proposed the donor oocyte model as a strategy to investigate the effects of characteristics such as endometrial receptivity on a cycle’s outcomes, as oocytes were often obtained from young donors with a good prognosis. However, except for the age, no other recipients’ characteristics affected the pregnancy result.

On the other hand, a very strong correlation between donors’ and recipients’ clinical outcomes was noted. Our evidence demonstrated that the chance of successfully achieving a pregnancy is drastically increased (more than 54 times) when the pregnancy result was positive in the donor cycle. In addition, the ROC curve analysis demonstrated that the donor’s implantation rate can predict recipient pregnancy with a 98.0% accuracy.

Since oocyte donors are usually young women with good a prognosis, and considering the emotional and economic burdens involved in assisted reproduction treatments, the abovementioned information may be extremely valuable for counselling patients of advanced age contemplating pregnancy.

To the best of our knowledge, for the first time in an egg-sharing donation program, a correlation between donors’ and recipients’ clinical outcomes was demonstrated. It could be argued that the population evaluated here differs from that in most other studies, in which oocytes are obtained from young fertile oocyte donors. Indeed, in Brazil, until very recently egg donation could not be conducted for profitable purposes; therefore, surplus oocytes were only obtained from patients that are undergoing IVF treatments, and the oocytes available for donation originated from infertile couples. This creates an interesting situation in which the functionality of oocytes derived from infertile couples could be analysed.

In conclusion, oocyte donor implantation and successful pregnancy rates, high-quality embryos rate, and blastocyst development rate predict pregnancy in the oocyte recipient cycle. The strong association in pregnancy success between donors and recipients, and the lack of correlation between donor characteristics and cycles’ outcomes, highlight the importance of the oocyte quality on the success of ICSI treatment.

## References

[r1] Abdalla HI, Brooks AA, Johnson MR, Kirkland A, Thomas A, Studd JW (1994). Endometrial thickness: a predictor of implantation in ovum recipients?. Hum Reprod.

[r2] Abdalla HI, Wren ME, Thomas A, Korea L (1997). Age of the uterus does not affect pregnancy or implantation rates; a study of egg donation in women of different ages sharing oocytes from the same donor. Hum Reprod.

[r3] Al-Ghamdi A, Coskun S, Al-Hassan S, Al-Rejjal R, Awartani K (2008). The correlation between endometrial thickness and outcome of in vitro fertilization and embryo transfer (IVF-ET) outcome. Reprod Biol Endocrinol.

[r4] Amui J, Check JH, Cohen R (2011). Successful twin pregnancy in a donor oocyte recipient despite a maximum endometrial thickness in the late proliferative phase of 4 mm. Clin Exp Obstet Gynecol.

[r5] Arce H, Velilla E, López-Teijón M (2015). Association between endometrial thickness in oocyte donation cycles and pregnancy success rates. Reprod Fertil Dev.

[r6] Baird DT, Collins J, Egozcue J, Evers LH, Gianaroli L, Leridon H, Sunde A, Templeton A, Van Steirteghem A, Cohen J, Crosignani PG, Devroey P, Diedrich K, Fauser BC, Fraser L, Glasier A, Liebaers I, Mautone G, Penney G, Tarlatzis B, ESHRE Capri Workshop Group (2005). Fertility and ageing. Hum Reprod Update.

[r7] Baker VL, Brown MB, Luke B, Conrad KP (2015). Association of number of retrieved oocytes with live birth rate and birth weight: an analysis of 231,815 cycles of in vitro fertilization. Fertil Steril.

[r8] Barker MA, Boehnlein LM, Kovacs P, Lindheim SR (2009). Follicular and luteal phase endometrial thickness and echogenic pattern and pregnancy outcome in oocyte donation cycles. J Assist Reprod Genet.

[r9] Barton SE, Missmer SA, Ashby RK, Ginsburg ES (2010). Multivariate analysis of the association between oocyte donor characteristics, including basal follicle stimulating hormone (FSH) and age, and IVF cycle outcomes. Fertil Steril.

[r10] Bellver J, Melo MA, Bosch E, Serra V, Remohi J, Pellicer A (2007). Obesity and poor reproductive outcome: the potential role of the endometrium. Fertil Steril.

[r11] Bellver J, Pellicer A, Garcia-Velasco JA, Ballesteros A, Remohi J, Meseguer M (2013). Obesity reduces uterine receptivity: clinical experience from 9,587 first cycles of ovum donation with normal weight donors. Fertil Steril.

[r12] Borini A, Bianchi L, Violini F, Maccolini A, Cattoli M, Flamigni C (1996). Oocyte donation program: pregnancy and implantation rates in women of different ages sharing oocytes from single donor. Fertil Steril.

[r13] Capelouto SM, Nagy ZP, Shapiro DB, Archer SR, Ellis DP, Smith AK, Spencer JB, Hipp HS (2018). Impact of male partner characteristics and semen parameters on in vitro fertilization and obstetric outcomes in a frozen oocyte donor model. Fertil Steril.

[r14] Check JH, Askari HA, Choe J, Baker A, Adelson HG (1993). The effect of the age of the recipients on pregnancy rates following donor-oocyte replacement. J Assist Reprod Genet.

[r15] Dain L, Bider D, Levron J, Zinchenko V, Westler S, Dirnfeld M (2013). Thin endometrium in donor oocyte recipients: enigma or obstacle for implantation?. Fertil Steril.

[r16] Demirol A, Gurgan T (2004). Effect of treatment of intrauterine pathologies with office hysteroscopy in patients with recurrent IVF failure. Reprod Biomed Online.

[r17] Dietterich C, Check JH, Choe JK, Nazari A, Lurie D (2002). Increased endometrial thickness on the day of human chorionic gonadotropin injection does not adversely affect pregnancy or implantation rates following in vitro fertilization-embryo transfer. Fertil Steril.

[r18] Dyer S, Chambers GM, de Mouzon J, Nygren KG, Zegers-Hochschild F, Mansour R, Ishihara O, Banker M, Adamson GD (2016). International Committee for Monitoring Assisted Reproductive Technologies world report: Assisted Reproductive Technology 2008, 2009 and 2010. Hum Reprod.

[r19] Garcia-Velasco JA, Isaza V, Caligara C, Pellicer A, Remohi J, Simón C (2003). Factors that determine discordant outcome from shared oocytes. Fertil Steril.

[r20] Gawecka JE, Marh J, Ortega M, Yamauchi Y, Ward MA, Ward WS (2013). Mouse zygotes respond to severe sperm DNA damage by delaying paternal DNA replication and embryonic development. PLoS One.

[r21] Hariton E, Kim K, Mumford SL, Palmor M, Bortoletto P, Cardozo ER, Karmon AE, Sabatini ME, Styer AK (2017). Total number of oocytes and zygotes are predictive of live birth pregnancy in fresh donor oocyte in vitro fertilization cycles. Fertil Steril.

[r22] Harris SE, Faddy M, Levett S, Sharma V, Gosden R (2002). Analysis of donor heterogeneity as a factor affecting the clinical outcome of oocyte donation. Hum Fertil (Camb).

[r23] Huang LS, Lee MS, Cheng EH, Lee TH, Liu CH, Lee MC, Chou MC (2008). Recipient age and pulsatility index affect uterine receptivity in oocyte donation programmes. Reprod Biomed Online.

[r24] Jungheim ES, Schon SB, Schulte MB, DeUgarte DA, Fowler SA, Tuuli MG (2013). IVF outcomes in obese donor oocyte recipients: a systematic review and meta-analysis. Hum Reprod.

[r25] Kasius A, Smit JG, Torrance HL, Eijkemans MJ, Mol BW, Opmeer BC, Broekmans FJ (2014). Endometrial thickness and pregnancy rates after IVF: a systematic review and meta-analysis. Hum Reprod Update.

[r26] Kuwayama M, Vajta G, Kato O, Leibo SP (2005). Highly efficient vitrification method for cryopreservation of human oocytes. Reprod Biomed Online.

[r27] Labarta E, Mariani G, Holtmann N, Celada P, Remohi J, Bosch E (2017). Low serum progesterone on the day of embryo transfer is associated with a diminished ongoing pregnancy rate in oocyte donation cycles after artificial endometrial preparation: a prospective study. Hum Reprod.

[r28] Levran D, Ben-Shlomo I, Dor J, Ben-Rafael Z, Nebel L, Mashiach S (1991). Aging of endometrium and oocytes: observations on conception and abortion rates in an egg donation model. Fertil Steril.

[r29] Li TC, Klentzeris L, Barratt C, Warren MA, Cooke S, Cooke ID (1993). A study of endometrial morphology in women who failed to conceive in a donor insemination programme. Br J Obstet Gynaecol.

[r30] Luke B, Brown MB, Stern JE, Missmer SA, Fujimoto VY, Leach R, SART Writing Group (2011). Female obesity adversely affects assisted reproductive technology (ART) pregnancy and live birth rates. Hum Reprod.

[r31] Lutjen P, Trounson A, Leeton J, Findlay J, Wood C, Renou P (1984). The establishment and maintenance of pregnancy using in vitro fertilization and embryo donation in a patient with primary ovarian failure. Nature.

[r32] Mac Conell EF, Almeida PG, Martins KE, Araújo JC, Chernicharo CA (2015). Bacterial community involved in the nitrogen cycle in a down-flow sponge-based trickling filter treating UASB effluent. Water Sci Technol.

[r33] Marcus Y, Segev E, Shefer G, Sack J, Tal B, Yaron M, Carmeli E, Shefer L, Margaliot M, Limor R, Gilad S, Sofer Y, Stern N (2016). Multidisciplinary Treatment of the Metabolic Syndrome Lowers Blood Pressure Variability Independent of Blood Pressure Control. J Clin Hypertens (Greenwich).

[r34] Margalioth EJ, Ben-Chetrit A, Gal M, Eldar-Geva T (2006). Investigation and treatment of repeated implantation failure following IVF-ET. Hum Reprod.

[r35] Meldrum DR (1993). Female reproductive aging--ovarian and uterine factors. Fertil Steril.

[r36] Moomjy M, Cholst I, Mangieri R, Rosenwaks Z (1999). Oocyte donation: insights into implantation. Fertil Steril.

[r37] Noci I, Borri P, Chieffi O, Scarselli G, Biagiotti R, Moncini D, Paglierani M, Taddei G. I (1995). Aging of the human endometrium: a basic morphological and immunohistochemical study. Eur J Obstet Gynecol Reprod Biol.

[r38] Oyesanya OA, Olufowobi O, Ross W, Sharif K, Afnan M (2009). Prognosis of oocyte donation cycles: a prospective comparison of the in vitro fertilization-embryo transfer cycles of recipients who used shared oocytes versus those who used altruistic donors. Fertil Steril.

[r39] Palermo GD, Colombero LT, Rosenwaks Z (1997). The human sperm centrosome is responsible for normal syngamy and early embryonic development. Rev Reprod.

[r40] Paulson RJ, Hatch IE, Lobo RA, Sauer MV (1997). Cumulative conception and live birth rates after oocyte donation: implications regarding endometrial receptivity. Hum Reprod.

[r41] Provost MP, Acharya KS, Acharya CR, Yeh JS, Steward RG, Eaton JL, Goldfarb JM, Muasher SJ (2016). Pregnancy outcomes decline with increasing recipient body mass index: an analysis of 22,317 fresh donor/recipient cycles from the 2008-2010 Society for Assisted Reproductive Technology Clinic Outcome Reporting System registry. Fertil Steril.

[r42] Rashidi BH, Sadeghi M, Jafarabadi M, Tehrani Nejad ES (2005). Relationships between pregnancy rates following in vitro fertilization or intracytoplasmic sperm injection and endometrial thickness and pattern. Eur J Obstet Gynecol Reprod Biol.

[r43] Remohi J, Gartner B, Gallardo E, Yalil S, Simon C, Pellicer A (1997). Pregnancy and birth rates after oocyte donation. Fertil Steril.

[r44] Stolwijk AM, Zielhuis GA, Sauer MV, Hamilton CJ, Paulson RJ (1997). The impact of the woman's age on the success of standard and donor in vitro fertilization. Fertil Steril.

[r45] Tapia-Pizarro A, Figueroa P, Brito J, Marin JC, Munroe DJ, Croxatto HB (2014). Endometrial gene expression reveals compromised progesterone signaling in women refractory to embryo implantation. Reprod Biol Endocrinol.

[r46] Tufan E, Elter K, Durmusoglu F (2004). Assessment of reproductive ageing patterns by hormonal and ultrasonographic ovarian reserve tests. Hum Reprod.

[r47] Valdes CT, Schutt A, Simon C (2017). Implantation failure of endometrial origin: it is not pathology, but our failure to synchronize the developing embryo with a receptive endometrium. Fertil Steril.

[r48] Weckstein LN, Jacobson A, Galen D, Hampton K, Ivani K, Andres J (1993). Improvement of pregnancy rates with oocyte donation in older recipients with the addition of progesterone vaginal suppositories. Fertil Steril.

[r49] Yeh JS, Steward RG, Dude AM, Shah AA, Goldfarb JM, Muasher SJ (2014). Pregnancy outcomes decline in recipients over age 44: an analysis of 27,959 fresh donor oocyte in vitro fertilization cycles from the Society for Assisted Reproductive Technology. Fertil Steril.

[r50] Zenke U, Chetkowski RJ (2004). Transfer and uterine factors are the major recipient-related determinants of success with donor eggs. Fertil Steril.

[r51] Zhang T, Li Z, Ren X, Huang B, Zhu G, Yang W, Jin L (2018). Endometrial thickness as a predictor of the reproductive outcomes in fresh and frozen embryo transfer cycles: A retrospective cohort study of 1512 IVF cycles with morphologically good-quality blastocyst. Medicine (Baltimore).

[r52] Zhao J, Zhang Q, Li Y (2012). The effect of endometrial thickness and pattern measured by ultrasonography on pregnancy outcomes during IVF-ET cycles. Reprod Biol Endocrinol.

[r53] Zhao J, Zhang Q, Wang Y, Li Y (2014). Endometrial pattern, thickness and growth in predicting pregnancy outcome following 3319 IVF cycle. Reprod Biomed Online.

